# Exploring Differentially Expressed Sperm miRNAs in Idiopathic Recurrent Pregnancy Loss and Their Association with Early Embryonic Development

**DOI:** 10.3390/ncrna10040041

**Published:** 2024-07-21

**Authors:** Ayushi Thapliyal, Anil Kumar Tomar, Sarla Naglot, Soniya Dhiman, Sudip Kumar Datta, Jai Bhagwan Sharma, Neeta Singh, Savita Yadav

**Affiliations:** 1 Department of Biophysics, All India Institute of Medical Sciences, New Delhi 110029, India; 2 Division of Reproductive, Child Health and Nutrition, Indian Council of Medical Research (ICMR), New Delhi 110029, India; 3 Department of Obstetrics and Gynaecology, All India Institute of Medical Sciences, New Delhi 110029, India; 4 Department of Laboratory Medicine, All India Institute of Medical Sciences, New Delhi 110029, India

**Keywords:** differential expression analysis, male contributory factors, microRNA, recurrent pregnancy loss, spermatozoa

## Abstract

The high incidence of idiopathic recurrent pregnancy loss (iRPL) may stem from the limited research on male contributory factors. Many studies suggest that sperm DNA fragmentation and oxidative stress contribute to iRPL, but their roles are still debated. MicroRNAs (miRNAs) are short non-coding RNAs that regulate various biological processes by modulating gene expression. While differential expression of specific miRNAs has been observed in women suffering from recurrent miscarriages, paternal miRNAs remain unexplored. We hypothesize that analyzing sperm miRNAs can provide crucial insights into the pathophysiology of iRPL. Therefore, this study aims to identify dysregulated miRNAs in the spermatozoa of male partners of iRPL patients. Total mRNA was extracted from sperm samples of iRPL and control groups, followed by miRNA library preparation and high-output miRNA sequencing. Subsequently, raw sequence reads were processed for differential expression analysis, target prediction, and bioinformatics analysis. Twelve differentially expressed miRNAs were identified in the iRPL group, with eight miRNAs upregulated (hsa-miR-4454, hsa-miR-142-3p, hsa-miR-145-5p, hsa-miR-1290, hsa-miR-1246, hsa-miR-7977, hsa-miR-449c-5p, and hsa-miR-92b-3p) and four downregulated (hsa-miR-29c-3p, hsa-miR-30b-5p, hsa-miR-519a-2-5p, and hsa-miR-520b-5p). Functional enrichment analysis revealed that gene targets of the upregulated miRNAs are involved in various biological processes closely associated with sperm quality and embryonic development.

## 1. Introduction

The actual number of conceptions that result in pregnancy failure is significantly higher than the clinically recognized rate of 12–15% [1,2]. Maintaining a pregnancy involves several intricate molecular processes, and any disruption in these processes may lead to miscarriage. The term “miscarriage” encompasses all pregnancy losses occurring from conception up to 24 weeks of gestation [3]. Two or more consecutive failed clinical pregnancies before 20 weeks of gestation are specifically termed recurrent pregnancy loss (RPL), a complex reproductive disorder affecting about 3% of couples of reproductive age planning to conceive [4,5,6]. Clinical investigations of RPL primarily focus on genetic factors, maternal age, antiphospholipid syndrome, uterine abnormalities, thrombophilia, hormonal and metabolic disorders, infections, autoimmune issues, semen quality, and lifestyle factors. However, in approximately 50% of cases, no underlying pathology is identified despite comprehensive clinical evaluations, classifying these cases as idiopathic RPL (iRPL) [6]. The idiopathic nature of these pregnancy losses causes significant trauma for affected couples, imposing a substantial financial burden and ultimately leading to anxiety and a sense of insecurity.

The high incidence of iRPL cases may largely be attributed to the fact that most clinical investigations focus exclusively on female factors. Surprisingly, the clinical evaluation of male partners in RPL cases is very limited, typically encompassing only semen analysis and karyotyping. While these tests are necessary, they are not conclusive. Over the years, researchers have sought to identify potential paternal factors contributing to iRPL. They have found that parental chromosomal abnormalities, aneuploidy in the developing embryo, sperm DNA fragmentation, and oxidative stress may contribute to RPL [7,8,9,10,11]. However, the significance of these factors in RPL remains inconclusive due to contradictory studies [12,13]. Recent proteomics studies on male partners of iRPL patients have unveiled differentially expressed sperm proteins associated with oxidative stress and sperm quality [14,15,16]. These findings suggest a significant molecular association between male fertility and pregnancy outcomes.

In the past decade, research into the role of non-coding RNAs in various molecular pathways and the identification of their downstream targets has significantly advanced our understanding of the pathophysiology of different diseases. Extensive research on sperm mRNA cargo has estimated that sperm delivers approximately 18,000 mRNA molecules to the embryo, underscoring the potential of male contributory factors in early embryonic development [17,18]. MicroRNAs (miRNAs) are endogenous non-coding small RNA molecules, about 22 nucleotides in length, that regulate more than 60% of protein-coding human RNA by either regulating gene expression or degrading mRNA. MiRNAs play a crucial role in various regulatory biological processes, and alterations in their expression are linked to the development of many diseases, including RPL [19,20]. Several recent studies have reported the differential expression of specific miRNAs in RPL [21,22,23,24,25]. A systematic review of 21 studies by Patronia et al. identified 75 statistically significant differentially expressed miRNAs (DEMs) in women affected by RPL, including miR-184, miR-520, miR-155/-5p, miR-100-5p, miR-146a/-5p, miR-21, miR-23a-3p, miR-92a/-3p, and miR-221-3p [19]. However, no comprehensive research has been conducted on paternal miRNAs in this context. Thus, this study was designed to identify dysregulated sperm miRNAs in male partners of confirmed iRPL patients. We believe that profiling sperm miRNAs and analyzing their differential expression will enable the identification of miRNAs linked to sperm quality, fertilization, and early embryonic development. This, in turn, will help us infer their role in the pathophysiology of iRPL.

## 2. Results

**Figure 1:**
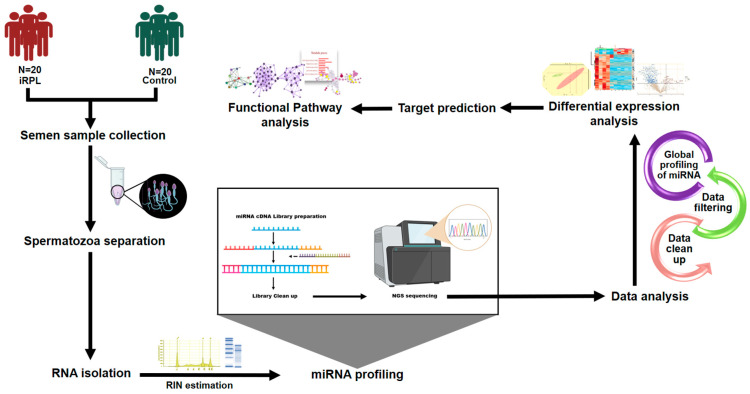
Study design.

An overview of the experimental setup is shown in Figure 1. After RNA extraction, a detailed gel analysis was performed to estimate the RNA integrity number (RIN) of 40 samples (*n* = 20 each, iRPL and controls). In iRPL samples, the RIN value ranged from 3.4 to 7.8, while in control samples, it ranged from 5 to 6.8. Based on their RIN values, samples were sorted separately in iRPL and control groups, and the top 10 samples from each group were further processed for miRNA library preparation and sequencing. The prepared miRNA libraries were cleaned up and assessed for total concentration and quality. In iRPL samples, cDNA concentration ranged from 3490 to 8370 pg/µL, while in controls, it ranged from 2630 to 5720 pg/µL. The raw sequence reads generated by sequencing were processed for the identification of sperm miRNAs and their differential expression analysis. Supplementary File S1 contains the details of raw and processed sequence reads.

### 2.1. Differentially Expressed miRNAs

**Figure 2:**
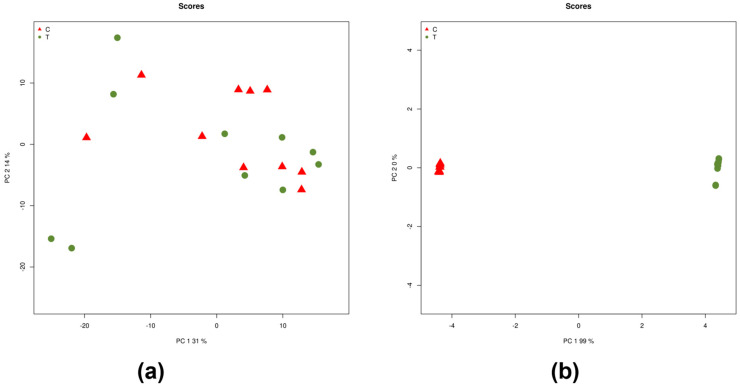
Principal component analysis considering (**a**) miRNA normalized counts and (**b**) batch corrected counts.

A total of 2656 miRNAs were identified from the raw sequence reads. Raw counts were obtained from mapped reads, and miRNAs with low counts were filtered out. The remaining 525 miRNAs with significant counts were processed for differential expression analysis (Supplementary File S2). The processed data files were subjected to principal component analysis (PCA) to determine data variance and identify differences between the groups. The PCA score plot based on normalized data did not show a separation between iRPL and control groups. However, the PCA score plot considering batch-corrected data showed a clear separation between the groups (Figure 2).

**Figure 3:**
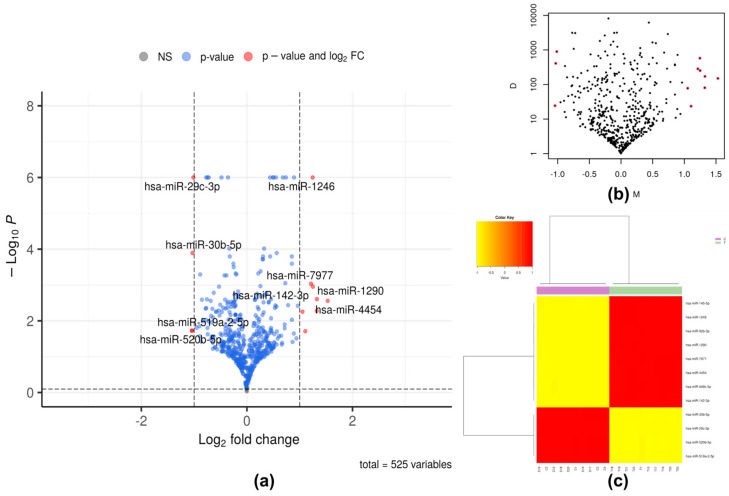
Differentially expressed microRNAs. (**a**) Volcano plot analysis with 1 and 0.8 cut-off values for log_2_ fold change (log_2_FC) and probability of differential expression (1-FDR), respectively; (**b**) mean difference plot representing the Log_2_FC (M) on the x-axis and absolute value of the difference between groups (D) on the y-axis. The significant differentially expressed miRNAs are highlighted in red dots; (**c**) heatmap, generated for significant differentially expressed miRNAs.

Twelve significant DEMs were identified, with eight being upregulated (hsa-miR-4454, hsa-miR-142-3p, hsa-miR-145-5p, hsa-miR-1290, hsa-miR-1246, hsa-miR-7977, hsa-miR-449c-5p, and hsa-miR-92b-3p) and four downregulated (hsa-miR-29c-3p, hsa-miR-30b-5p, hsa-miR-519a-2-5p, and hsa-miR-520b-5p) in the iRPL group compared to the control group (Table 1, Figure 3).

### 2.2. MicroRNA Gene Targets

To gain functional insights into the role of DEMs in sperm physiology contributing to iRPL, their gene targets were identified by miRWalk. With a binding score of 0.95, a total of 27,009 and 9366 binding sites at the 3′UTR region were retrieved for upregulated and downregulated miRNAs, respectively (Supplementary Files S2 and S3). The predicted targets with a seed score of 1 (i.e., perfectly matched gene-miRNA pair) were then sorted, resulting in 14,988 targets for upregulated miRNAs and 5101 targets for downregulated miRNAs. Next, multiple entries for the same genes were merged to generate the final list of miRNA-mRNA pairs, reducing the number of gene targets to 5661 for upregulated miRNAs and 1545 for downregulated miRNAs. For upregulated miRNAs, 422 validated target sites corresponding to 208 unique genes were identified. Similarly, 46 validated target sites corresponding to 24 unique genes were identified for downregulated miRNAs. For gene enrichment and pathway analysis, we selected miRNA gene targets identified by miRWalk and confirmed them by at least one of the following databases: TargetScan, miRDB, or miRTarBase. This approach yielded 768 gene targets for upregulated miRNAs and 235 gene targets for downregulated miRNAs (Supplementary Files S3 and S4).

### 2.3. Functional Annotations and Pathway Analysis

**Figure 4:**
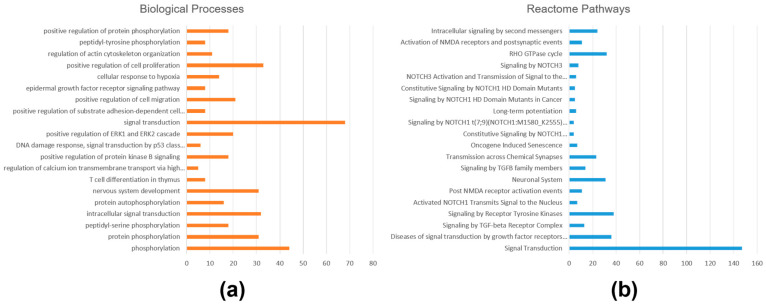
Functional enrichment and pathway analysis of gene targets of upregulated miRNAs in iRPL. (**a**) Biological processes enriched; (**b**) Reactome pathways overrepresented.

Gene enrichment and pathway overrepresentation analyses were performed separately for gene targets of upregulated and downregulated miRNAs in iRPL. For the gene targets of upregulated miRNA, a total of 228 GO terms (131 biological processes, 55 cellular components, and 42 molecular functions) were enriched, and 80 Reactome pathways were overrepresented with a *p*-value ≤ 0.05 (Supplementary File S5). Important GO terms that were enriched were protein phosphorylation, intracellular signal transduction, regulation of calcium ion transmembrane transport, positive regulation of protein kinase B signaling, DNA damage response, positive regulation of cell migration, epidermal growth factor receptor signaling pathway, positive regulation of cell proliferation, regulation of actin cytoskeleton organization (biological processes), protein binding, protein tyrosine kinase activity, integrin binding, small GTPase binding, growth factor binding, ubiquitin-protein transferase activity, ATP binding, and RNA polymerase II transcription factor activity (molecular functions); and crucial pathways that were overrepresented were signal transduction, signaling by TGF-beta receptor complex, NOTCH1 signaling, RHO GTPase cycle, and intracellular signaling by second messengers. The top 20 enriched biological processes and overrepresented Reactome pathways are shown in Figure 4.

**Figure 5:**
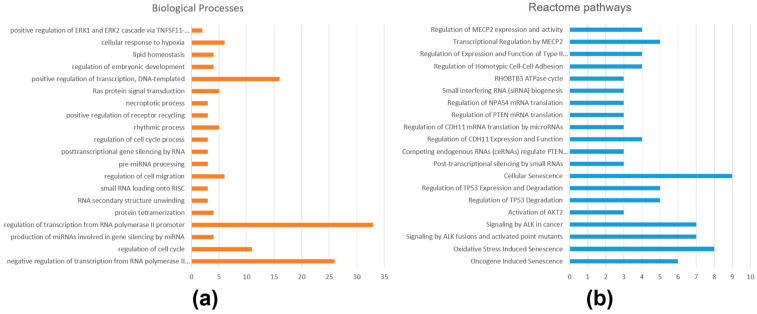
Functional enrichment and pathway analysis of gene targets of downregulated miRNAs in iRPL. (**a**) Biological processes enriched; (**b**) Reactome pathways overrepresented.

For the gene targets of downregulated miRNAs, only 60 GO terms (32 biological processes, 12 cellular components, and 16 molecular functions) were enriched, and 52 Reactome pathways were overrepresented with a *p*-value ≤ 0.05 (Supplementary File S6). The list of enriched GO terms included regulation of transcription from RNA polymerase II promoter, regulation of cell cycle, production of miRNAs involved in gene silencing by miRNA, posttranscriptional gene silencing by RNA, regulation of embryonic development (biological processes), protein binding, actin binding, DNA binding, miRNA binding, transcription corepressor activity, translation initiation factor activity, and histone deacetylase regulator activity (molecular functions). Similarly, oxidative stress-induced senescence, activation of AKT2, regulation of TP53 expression and degradation, post-transcriptional silencing by small RNAs, small interfering RNA (siRNA) biogenesis, regulation of homotypic cell–cell adhesion, and transcriptional regulation by MECP2 pathways were overrepresented. The top 20 enriched biological processes and overrepresented Reactome pathways are shown in Figure 5.

### 2.4. Protein–Protein Interaction Networks

**Figure 6:**
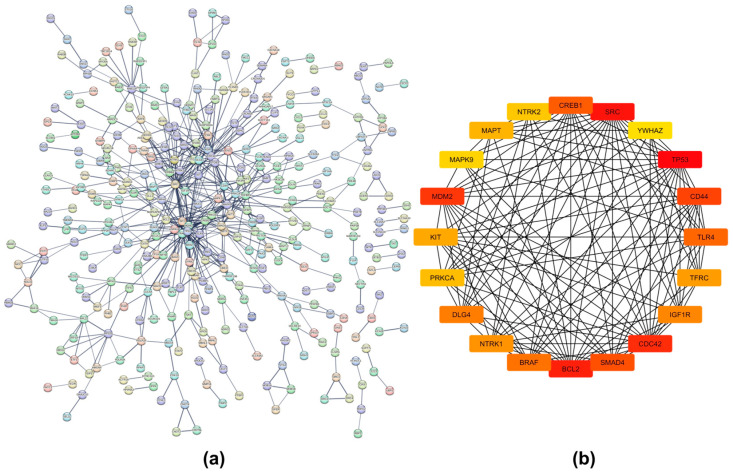
Network analysis of gene targets of upregulated miRNAs in iRPL. (**a**) Protein–protein interaction (PPI) network generated by STRING database; (**b**) top 20 hub genes identified by Cytoscape.

**Figure 7:**
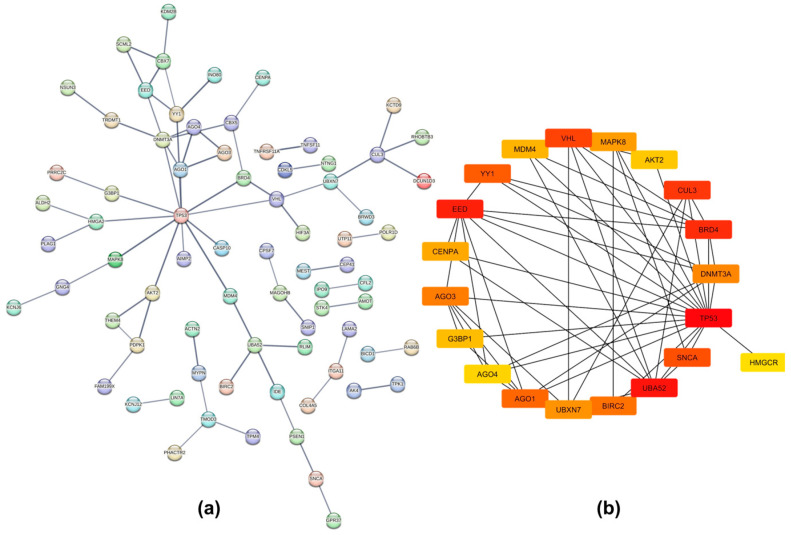
Network analysis of gene targets of downregulated miRNAs in iRPL. (**a**) Protein–protein interaction (PPI) network generated by STRING database; (**b**) top 20 hub genes identified by Cytoscape.

A dense protein–protein interaction (PPI) network was generated for proteins translated from gene targets of upregulated miRNAs in iRPL (nodes = 767, edges = 597, average node degree = 1.56, and PPI enrichment *p*-value = 1.04 × 10^−9^) with a high confidence interaction score (minimum score = 0.7) (Figure 6a). On the other hand, the PPI network of proteins translated from gene targets of downregulated miRNAs had 234 nodes, 71 edges, an average node degree of 0.607, and a PPI enrichment *p*-value of 0.00143 (Figure 7a). The raw networks, with a minimum interaction score of 0.4, were imported into Cytoscape to identify hub genes using the cytoHubba application.

The top 20 hub nodes identified in the network of gene targets of upregulated miRNAs were cyclic AMP-responsive element-binding protein 1 (CREB1), tyrosine-protein kinase Src (SRC), 14-3-3 protein zeta/delta (YWHAZ), tumor antigen p53 (TP53), CD44 antigen (CD44), toll-like receptor 4 (TLR4), transferrin receptor protein 1 (TFRC), insulin-like growth factor 1 receptor alpha chain (IGF1R), cell division control protein 42 homolog (CDC42), mothers against decapentaplegic homolog 4 (SMAD4), apoptosis regulator Bcl-2 (BCL2), serine/threonine-protein kinase B-raf (BRAF), high-affinity nerve growth factor receptor (NTRK1), disks large homolog 4 (DLG4), protein kinase C alpha type (PRKCA), mast/stem cell growth factor receptor Kit (KIT), E3 ubiquitin-protein ligase Mdm2 (MDM2), mitogen-activated protein kinase 9 (MAPK9), microtubule-associated protein tau (MAPT), and BDNF/NT-3 growth factors receptor (NTRK2) (Figure 6b). Those identified in the network of gene targets of downregulated miRNAs were Von Hippel-Lindau disease tumor suppressor (VHL), mitogen-activated protein kinase 8 (MAPK8), AKT Serine/Threonine Kinase 2 (AKT2), cullin-3 (CUL3), bromodomain-containing protein 4 (BRD4), DNA methyltransferase 3 alpha (DNMT3A), TP53, 3-hydroxy-3-methylglutaryl-coenzyme A reductase (HMGCR), alpha-synuclein (SNCA), ubiquitin-60S ribosomal protein L40 (UBA52), Baculoviral IAP repeat-containing protein 2 (BIRC2), UBX domain-containing protein 7 (UBXN7), argonaute RISC Components (AGO1, AGO3, and AGO4), ras GTPase-activating protein-binding protein 1 (G3BP1), histone H3-like centromeric protein A (CENPA), polycomb protein embryonic ectoderm development (EED), YY1 transcription factor (YY1), and protein Mdm4 (MDM4) (Figure 7b).

### 2.5. MicroRNA Expression Confirmation by Quantitative RT-PCR

Quantitative RT-PCR experiments were conducted to validate the expression of 12 DEMs. The expression patterns of 11 miRNAs were consistent with the differential expression data obtained from high-throughput sequencing (Table 1). However, no significant difference was observed in the expression of one of the miRNAs, hsa-miR-30b-5p, between the iRPL and control samples (FC = 0.971; *p*-value = 0.4853). Among the 11 concordant miRNAs, all upregulated miRNAs exhibited significant expression changes, with *p*-values ranging between 0.0183 and 0.0538. In contrast, the two downregulated miRNAs, hsa-miR-29c-3p (FC = 0.056; *p*-value = 0.1755) and hsa-miR-520b-5p (FC = 0.374; *p*-value = 0.2469), had higher *p*-values, indicating less statistical significance in their expression differences.

## 3. Discussion

Many studies have confirmed the role of spermatozoa in post-fertilization processes and embryonic development, encouraging researchers to investigate paternal factors to elucidate the molecular mechanisms underlying iRPL [26,27]. In recent years, paternal factors such as DNA alterations, sperm proteomic changes, and reactive oxygen species (ROS) levels leading to poor sperm quality have been extensively studied [15,28,29,30,31]. Furthermore, differential sperm proteomics studies have revealed the altered expression of proteins associated with various biological processes such as oxidative stress, stress response, and protein folding/refolding, in iRPL [14,15,16,32]. However, the role of male contributory factors and the associated biological processes leading to iRPL remain inadequately understood, underscoring the necessity for integrative molecular studies to achieve a comprehensive understanding of iRPL. Spermatozoa retain various RNA species, some of which are transferred to the oocyte during fertilization [17,33]. This suggests that in addition to sperm quality, post-transcriptional and translational processes may also influence post-fertilization events and early embryonic development. Thus, molecular alterations in spermatozoa may potentially hold the key to unraveling the pathophysiology of idiopathic cases of RPL.

Recent advancements in next-generation sequencing technology have enabled scientists to investigate non-coding RNAs in various clinical samples, identifying changes in their expression and their implications for disease progression. Maternal miRNAs and lnc-RNAs have been implicated in regulating critical aspects of pregnancy, including embryo implantation and RPL [20,34]. Also, altered expression levels of miRNAs in testicular tissue, human sperm, and seminal plasma have been associated with various subfertility issues [35,36,37]. Interestingly, Wu et al. reported that sperm miRNAs in bulls play a crucial role in embryonic development [38]. However, analogous studies in humans are lacking, and the role of human sperm miRNAs in iRPL remains unexplored. Therefore, we designed this study to identify sperm miRNAs and their differential expression in iRPL cases, aiming to elucidate the underlying molecular mechanisms that may contribute to multiple miscarriages in these cases.

In this study, 12 significant DEMs with a |log_2_FC| > 1 were identified in the spermatozoa of iRPL cases compared to fertile controls. While hsa-miR-4454, hsa-miR-142-3p, hsa-miR-145-5p, hsa-miR-1290, hsa-miR-1246, hsa-miR-7977, hsa-miR-449c-5p, and hsa-miR-92b-3p were upregulated, hsa-miR-29c-3p, hsa-miR-30b-5p, hsa-miR-519a-2-5p, and hsa-miR-520b-5p were found to be downregulated. Among these DEMs, hsa-miR-1290, hsa-miR-145-5p, and hsa-miR-7977 have been previously identified as having a significant role in semen quality. Cao et al. reported that FOXP3 activates the transcription of linc00857, which in turn increases the GPX4 mRNA stability via sponging hsa-miR-1290, thereby inhibiting ferroptosis [39]. On searching miRWalk, it was found that GPX4 is a gene target of hsa-miR-1290 (score = 0.846). Interestingly, reduced GPX4 expression in spermatozoa samples from male partners of iRPL patients was also observed in one of our previous studies [16]. This reduction likely impairs the spermatozoa’s ability to defend against the detrimental effects of lipid peroxidation, significantly impacting sperm quality. The overexpression of has-miR-1290, as observed in this study, might cause the downregulation of GPX4 mRNA and encoded protein leading to the accumulation of lipid peroxidation by products, thus contributing to iRPL. The expression of hsa-miR-145-5p has been directly linked to sperm quality. In idiopathic infertile men, the overexpression of hsa-miR-145-5p in spermatozoa results in decreased levels of its target, MLH1 mRNA, and is associated with increased seminal oxidative stress and sperm DNA fragmentation [40]. miRNAs are pivotal in influencing age-related changes in male fertility and may provide valuable insights into the mechanisms driving the decline in fertility as men age. Zhao et al. analyzed changes in miRNA expression profiles of human spermatozoa and discovered that hsa-miR-7977 expression increases with age [41]. This finding suggests that elevated hsa-miR-7977 levels may be linked to poor sperm quality. Thus, the increased expression of hsa-miR-7977 in iRPL cases, as observed in this study, implies that the expression of its gene targets is crucial for sperm quality. Furthermore, these genes likely play a vital role in post-fertilization processes and embryonic development. A significant DEM identified in this study, hsa-miR-449c-5p, is known for its crucial role in various processes, including brain development, motile ciliogenesis, spermatogenesis, growth of gastric carcinoma, and the migration of liver cancer cells by targeting the post-transcriptional regulation of genes through both extracellular signal and intracellular pathways [42,43,44,45]. It has been reported that hsa-miR-1246 regulates two important pathways, calcium metabolism and the regulation of stem cell differentiation [46]. These pathways are closely associated with embryonic development [47,48]. Overexpression of this miRNA in iRPL spermatozoa suggests a potential role in embryonic development. Another notable DEM, hsa-miR-92b-3p is recognized for its regulatory roles in cell cycle and apoptosis, and its abnormal expression has been extensively observed across various types of tumors [49,50,51,52]. It modulates the expression of its target proteins, including four and a half LIM domains protein 2 (FHL2), glutathione-S-transferase M3 (GSTM3), and cyclin-dependent kinase inhibitor 1 (CDKN1A), and it eventually affects various signaling pathways [53,54]. While not conclusive, a few studies have reported dysregulated expression of hsa-miR-92b-3p in reproductive physiology. Heidary et al. reported the altered expression of hsa-miR-92b-3p in the spermatozoa of men with unexplained asthenozoospermia; however, they observed no significant variation in quantitative PCR validation [55]. The altered expression of maternal uterine fluid miR-92b-3p has been linked to recurrent implantation failure [56].

To gain insight into the functional roles of the altered miRNAs, their target genes were predicted. Subsequently, these target genes were investigated to elucidate their biological significance using the bioinformatics tools of functional annotation and pathway analysis. The gene targets of upregulated miRNAs were associated with various signaling pathways, including TGF-beta receptor complex, tyrosine kinases, NOTCH1-3, NTRKs, ERKs, VEGF, MAPK family, PI3K/AKT, rho GTPases, and ephrin; and they were also associated with biological processes, such as protein phosphorylation, regulation of cell migration and proliferation, actin cytoskeleton organization, calcium ion import across the plasma membrane, regulation of transcription, regulation of transcription, embryonic hemopoiesis, multicellular organism development, DNA damage response, regulation of signaling pathways, and cellular metabolic processes. Most of these pathways and biological processes are linked, either directly or indirectly, to the structure, motility, and quality of spermatozoa, as well as to post-fertilization events and early embryonic development. The important proteins encoded by gene targets of upregulated miRNAs include ADAM metallopeptidase domain 17 (ADAM17), AKT2, AKT3, apolipoprotein B mRNA editing enzyme catalytic subunit 3F (APOBEC3F), BCL2 apoptosis regulator (BAHD1), beta-1,3-galactosyltransferase 5 (ATXN7L1), DnaJ heat shock protein family (HSP40) member B6 (DENND4B), heat shock protein family A (HSP70) member 1B (HMBOX1), matrix metallopeptidase 16 (MFSD8), and calcium/calmodulin-dependent protein kinase IV (CAMK4); while those encoded by targets of downregulated miRNA included DNA methyltransferase 3 alpha (DNMT3A), EED, nuclear autoantigenic sperm protein (NASP), and hypoxia-inducible factor 3 subunit alpha (HIF3A). In spermatozoa, these proteins are responsible for sperm quality and are also associated with early embryonic development.

AKT3, a member of the AKT protein kinase family, plays a crucial role in regulating a wide range of cellular functions. AKT3 induces oxidative stress and DNA damage by upregulating reactive oxygen species (ROS) within cells, and high levels of AKT3 inactivate the DNA damage response [57]. The target prediction data show that two of the upregulated miRNAs, hsa-miR-142-3p and hsa-miR-145-5p, bind to AKT3. Their upregulation in iRPL cases can be interpreted as a response by spermatozoa to counteract the oxidative stress and DNA damage induced by AKT3. This finding aligns with proteomics studies that indicate increased oxidative stress in iRPL spermatozoa, which may contribute to reduced sperm quality. This reduction is attributed to the downregulated expression of proteins associated with the oxidative stress response and DNA damage repair [14,15,16]. Heat shock proteins (HSPs) are abundantly present in spermatozoa and are crucial for various sperm functions, including sperm vitality, motility, and capacitation [58,59,60,61]. Additionally, miRNAs regulate the expression of specific HSPs, including HSP40, HSP70, and HSP90, in human sperm [62]. This regulatory mechanism significantly influences sperm function. Interestingly, miRNAs specific to the HSP40 and HSP70 family members, hsa-miR-145-5p and hsa-miR-449c-5p, were found to be upregulated in this study. This implies that the regulation of stress response proteins by these miRNAs might lead to poor sperm quality in iRPL. Calcium/calmodulin-dependent protein kinases are key regulators of various molecular functions within the calcium-activated CaMKK-CaMK4 signaling cascade. A recent study conducted on a mouse model of abortion has shown that CaMK4 is essential for the regulation of Th17 cell infiltration and associated cytokine production at the maternal–fetal interface through the AKT/mTOR pathway [63]. ADAM17 and EED proteins are closely associated with the molecular processes involved in embryo development. ADAM17, a matrix metalloprotease, is involved in the physiological apoptosis of germ cells and plays a significant role in spermatogenesis [64]. Other members of the ADAMs protein family are known to participate in various biological processes, including fertilization, myogenesis, neurogenesis, heart development, and endothelial permeability. EED is a component of polycomb histone-modifying repressor complexes that silence transcription factors specific to particular cell types, preventing their expression outside of appropriate spatial domains. Mutations in these proteins frequently result in aberrant gene expression, developmental defects, and embryonic lethality [65]. The gene targets of dysregulated miRNAs encode crucial proteins necessary for the structural integrity and function of spermatozoa, as well as for post-fertilization events and key developmental processes.

Further, gene enrichment and pathway analysis also suggest that dysregulated miRNAs might influence many biological processes associated with sperm quality and functions by regulating the expression of genes essential for sperm morphology, motility, and capacitation, including protein phosphorylation, intracellular signal transduction, regulation of calcium ion transmembrane transport, DNA damage response, positive regulation of cell migration, epidermal growth factor receptor signaling pathways, and the regulation of actin cytoskeleton organization. This can adversely impact fertilization, as well as post-fertilization processes. Similar conclusions were reached through pathway analysis. The PPI networks showed that miRNA targets are interconnected both physically and functionally. Most of the hub genes identified in the network of upregulated miRNA gene targets are involved in spermatogenesis, sperm functions, and related processes. For example, CREB1, SRC, YWHAZ, TP53, TFRC, IGF1R, KIT, MDM2, and MAPK9 are involved in spermatogenesis [66,67,68,69,70,71,72]. TP53 also helps in the elimination of defective sperm cells and contributes to sperm quality [69]. While PRKCA participates in sperm capacitation and acrosome reaction, CD44 helps in sperm binding to the oviduct or ovum itself [73,74]. CDC42 is essential for normal sperm development and acrosome reaction [75]. It is involved in the cytoskeleton organization of sperm and the formation of the sperm tail and head. Another hub gene, BCL2, is an apoptosis inhibitor that protects sperm cells by minimizing ROS production. This is crucial for the efficient removal of defective sperm cells, ensuring proper sperm development and quality [76]. Thus, the suppression of these genes by miRNAs could impair sperm quality, leading to various fertilization and post-fertilization defects. Such disruptions can adversely affect normal embryonic development and contribute to pregnancy loss in cases of iRPL.

The expression of DEMs was also validated in iRPL and control samples using quantitative RT-PCR. Except for hsa-miR-30b-5p, the expression patterns of the remaining 11 miRNAs were consistent with the high-throughput sequencing data. Also, higher *p*-values were observed for two miRNAs, hsa-miR-29c-3p and hsa-miR-520b-5p. These findings validate alterations in the expression of most miRNAs, but the lack of significant expression changes for hsa-miR-30b-5p and the higher *p*-values for hsa-miR-29c-3p and hsa-miR-520b-5p warrant further investigation.

## 4. Materials and Methods

### 4.1. Recruitment of Study Participants

This study was approved by the Institutional Ethics Committee of the All India Institute of Medical Sciences (AIIMS), New Delhi (IEC-158/04.03.2022, RP-46/2022). The study participants, male partners of iRPL patients and controls, were recruited from the Department of Obstetrics and Gynaecology, AIIMS, New Delhi. The following were the inclusion and exclusion criteria for the recruitment of study participants.

*Inclusion criteria for the iRPL group:*

- Male partners of confirmed iRPL women with a history of 2 or more spontaneous abortions in the clinical first trimester
- Age < 45 years
- Normal semen profile
- No history of autoimmune or endocrine disorder
- Clinical workup should have ruled out maternal RPL etiologies, including genetic, anatomic, immunological factors, inherited thrombophilia, infections, environmental and lifestyle factors, birth defects in families, heart disease, diabetes, etc.

*Inclusion criteria for the control group:*

- Age-matched volunteers who have fathered a healthy child within the last 2 years.
- Normal semen profile
- No history of RPL in female partner

*Exclusion criteria:*

- Participants who did not meet the inclusion criteria
- Male partners of iRPL patients who had abnormal karyotypes, endocrine problems, immunological disorders, or any other significant illnesses.
- Semen samples that exhibited abnormal semen parameters or a substantial presence of round cells

After recruitment, participants were asked to sign an informed consent form, and semen samples were collected from those who signed the form.

### 4.2. Semen Collection and Analysis

Semen samples were obtained through masturbation following a period of sexual abstinence lasting 4–7 days. Semen samples were first allowed to liquefy at room temperature for 30 min. After liquefaction, semen assessment was performed according to the World Health Organization (WHO) laboratory manual for the examination and processing of human semen (6th ed., 2021) in the Department of Laboratory Medicine, AIIMS, New Delhi. Based on semen parameters, 60 samples (*n* = 30 for each, iRPL and controls) were included in the study (Table 2).

### 4.3. Separation of Spermatozoa

Spermatozoa were separated from seminal plasma using a two-layered Percoll gradient method [77]. After equilibration, the upper (45%) and lower phases (90%) of Percoll prepared using HAM’s F10 medium were transferred to a 15 mL sterile falcon tube. The liquefied semen was layered over the upper phase, followed by centrifugation at 500 g for 30 min. After removal of the supernatant, the sperm pellet was washed three times with phosphate buffer saline and stored in Trizol at −80 °C for further experiments.

### 4.4. RNA Extraction

Total RNA was extracted from 40 spermatozoa samples using a total RNA extraction kit (RNeasy Mini Kit, Qiagen, Hilden, Germany) following the manufacturer’s protocol. After extraction, RNA concentration was determined in each sample with the NanoDrop ND-100 Spectrophotometer (NanoDrop Technologies, Wilmington, DE, USA) and Qubit 4 Fluorometer (Thermo Fisher Scientific—Indianapolis, IN, USA). RNA quality was assessed by a high-throughput automated electrophoresis platform, Tapestation 4200™ (Agilent Technologies, Santa Clara, CA, USA). The 4200 TapeStation Software displays the results as an electropherogram, a gel image, and the RIN value.

### 4.5. MicroRNA Library Preparation and Sequencing

Human sperm miRNA libraries were prepared for 20 samples (*n* = 10 each, iRPL and controls) with an initial RNA input of 200 ng. Adapters were ligated sequentially to the 3′ and 5′ ends of miRNAs in an unbiased reaction, followed by universal cDNA synthesis with the assignment of unique molecular identifiers (UMIs). Subsequently, cDNA clean-up, library amplification, and library clean-up were performed. The size profile and quality of miRNA libraries were assessed using a high-sensitivity D1000 ScreenTape^®^ on a Tapestation 4200™ system. The prepared libraries were sequenced on the Illumina Nextseq 2K platform (Illumina, San Diego, CA, USA) to generate 15 million 1 × 150 bp reads per sample. The sequenced data were processed to generate FASTQ files. The sequence reads were first processed to extract the UMI barcode using UMI tools, and adapters were removed by Trim Galore. The processed sequence reads were collapsed to obtain unique sequences, with each unique collapsed sequence annotated with its corresponding read counts. The quality of the generated sequence data was evaluated using the FastQC tool (version 0.11.9). The alignment was performed to the reference genome assembly (hg38, GRCh38.p12) and reference miRNA (miRBase Release 22.1) using the miRDeep2 tool [78].

### 4.6. Expression Estimation of Sperm miRNAs

Raw counts for each miRNA were obtained from the mapped reads, and miRNAs with an average count per million (CPM) less than 1 per condition were excluded to mitigate noise. Normalization was performed with the trimmed mean of M values (TMM) method, which is an inter-sample normalization method [79]. TMM normalization equalizes the total RNA output across samples without factoring in gene length or library size. The hidden batch effect was corrected with the ARSyNseq function of the NOISeq R package. Principal component analysis (PCA) was performed to validate the unknown batch removal correction by visualizing the clustering of the multivariate data. Differential miRNA expression analysis was performed by the NOISeq R package, and the differential expression profile was shown by mean difference, volcano, and heatmap plots. The cut-off values for log_2_ fold change (log_2_FC) and the probability of differential expression (1-FDR) were set as 1 and 0.8, respectively, to identify significant DEMs.

### 4.7. MicroRNA Target Prediction

Gene targets for the significant DEMs were predicted using an open-source platform, miRWalk version 2 [80]. It employs the TarPmiR program utilizing a random forest-based approach for predicting miRNA target sites. It also integrates miRNA-target interaction results from three other databases—TargetScan, miRDB, and miRTarBase. While TargetScan and miRDB provide predicted interactions, miRTarBase offers validated data sets on miRNA-mRNA interactions. For each miRNA, binding sites at the 3′UTR region were retrieved with a binding score of 0.95, followed by the selection of targets with a seed score of 1. Potential gene targets were predicted for upregulated and downregulated miRNAs separately.

### 4.8. Bioinformatics Analysis

To understand the biological significance of the identified gene targets of significant DEMs in the iRPL pathophysiology, functional annotations and pathway analysis were performed using the DAVID gene enrichment tool v2021, while their interaction networks were assessed by STRING database v12 and hub genes were identified by the Cytoscape tool v3.10.1 [81,82].

### 4.9. MicroRNA Expression Analysis by Quantitative RT-PCR

The expression of DEMs was validated by quantitative RT-PCR using a CFX 96 thermocycler (Bio-Rad). The cDNAs were synthesized from 20 samples (*n* = 10 each, iRPL and control groups) using the miRCURY LNA^®^ RT Kit (Qiagen, Duesseldorf, Germany), following the manufacturer’s instructions. The reverse transcription reaction mixture was incubated for 60 min at 42 °C, followed by the inactivation of the reverse transcriptase enzyme at 95 °C. The resulting cDNA was then stored at −20 °C until further use. Primers for the selected miRNAs and the U6 reference gene were designed using sRNAPrimerDB, a web-based primer design tool specifically for small non-coding RNAs [83]. Primer pairs were chosen for their specificity and optimized for the respective miRNA (Table S1). Real-time PCR experiments were conducted utilizing the miRCURY LNA SYBR^®^ Green PCR Kit (Qiagen, Duesseldorf, Germany), following the manufacturer’s instructions. The PCR program was executed as specified: initial denaturation at 95 °C for 2 min, followed by 45 cycles of 2-step amplification, including denaturation at 95 °C for 10 s and annealing at the respective Tm for 60 s. The relative changes in the expression of the miRNAs were determined using the ΔΔCt method.

## 5. Conclusions

The global miRNA profiling combined with bioinformatics analysis conducted in this study provides a comprehensive overview of differentially abundant miRNAs in iRPL. It suggests that potential target genes of these miRNAs are closely associated with sperm quality and early embryonic development processes. Functional enrichment analysis revealed their involvement in several important biological processes, including protein phosphorylation, developmental processes, nervous system development, intracellular signal transduction, and the regulation of various signaling pathways. These findings offer a foundation for future research to understand how dysregulated miRNAs in spermatozoa might contribute to iRPL, enhancing our knowledge of male contributory factors in idiopathic miscarriages.

## Figures and Tables

**Table 1:** Differentially expressed microRNAs in idiopathic recurrent pregnancy loss (iRPL).

	High-Throughput Sequencing			Quantitative RT-PCR	
miRNA	Fold Change (FC)	Log_2_ (FC)	Probability (1-FDR)	FC	*p*-Value
*Upregulated in iRPL*					
hsa-miR-4454	2.888	1.530	0.997	6.487	0.0196
hsa-miR-142-3p	2.505	1.325	0.998	69.824	0.0538
hsa-miR-145-5p	2.501	1.322	0.995	14.070	0.0213
hsa-miR-1290	2.377	1.249	0.999	20.551	0.0405
hsa-miR-1246	2.371	1.245	1.000	109.297	0.0183
hsa-miR-7977	2.319	1.213	0.999	8.282	0.0525
hsa-miR-449c-5p	2.153	1.106	0.981	13.951	0.0389
hsa-miR-92b-3p	2.074	1.053	0.994	17.185	0.0310
*Downregulated in iRPL*					
hsa-miR-29c-3p	0.495	−1.015	1.000	0.056	0.1755
hsa-miR-30b-5p	0.489	−1.031	1.000	0.971	0.4853
hsa-miR-519a-2-5p	0.485	−1.043	0.981	0.114	0.0476
hsa-miR-520b-5p	0.485	−1.043	0.981	0.374	0.2469

**Table 2:** Sample details.

	High-Throughput Sequencing		Quantitative RT-PCR	
Features	iRPL (*n* = 20) *	Controls (*n* = 20) *	iRPL (*n* = 10) *	Controls (*n* = 10) *
Age of donors (years)	29.05 ± 3.2	32.0 ± 1.6	30.2 ± 3.7	30.6 ± 3
Number of abortions in female partners	4.15 ± 1.1	-	3.8 ± 2.0	-
Sample volume (mL)	2.72 ± 0.8	3.2 ± 0.9	3.1 ± 1.2	3.5 ± 1.3
Sperm Count (million/mL)	76.40 ± 44.5	117.30 ± 65.2	96.1 ± 25.1	113.6 ± 59.5
Sperm Motility (%)	71 ± 9.5	69.5 ± 8.4	63 ± 8.8	69.5 ± 7.21

* All parameters are shown as value ± SD.

## Data Availability

The raw sequence data files that support the findings of this study are available from the corresponding author upon reasonable request. All other files generated are provided as Supplementary Files.

## Supplementary Materials

The following supporting information can be downloaded at https://www.mdpi.com/article/10.3390/ncrna10040041/s1, Supplementary File S1: Details of raw and processed sequence reads; Supplementary File S2: MicroRNA counts in iRPL and control samples; Supplementary File S3: Gene targets of upregulated miRNAs in iRPL, predicted by miRWalk; Supplementary File S4: Gene targets of downregulated miRNAs in iRPL, predicted by miRWalk; Supplementary File S5: GO terms and Reactome pathways enriched among gene targets of upregulated miRNAs in iRPL; Supplementary File S6: GO terms and Reactome pathways enriched among gene targets of downregulated miRNAs in iRPL; Table S1: List of primers.

## References

[1] Jeve Y.B., Davies W. (2014). Evidence-based management of recurrent miscarriages. J. Hum. Reprod. Sci..

[2] Wilcox A.J., Weinberg C.R., O’Connor J.F., Baird D.D., Schlatterer J.P., Canfield R.E., Armstrong E.G., Nisula B.C. (1988). Incidence of early loss of pregnancy. N. Engl. J. Med..

[3] Rai R., Regan L. (2006). Recurrent miscarriage. Lancet.

[4] Berry C.W., Brambati B., Eskes T.K., Exalto N., Fox H., Geraedts J.P., Gerhard I., Gonzales Gomes F., Grudzinskas J.G., Hustin J. (1995). The Euro-Team Early Pregnancy (ETEP) protocol for recurrent miscarriage. Hum. Reprod..

[5] Ford H.B., Schust D.J. (2009). Recurrent pregnancy loss: Etiology, diagnosis, and therapy. Rev. Obstet. Gynecol..

[6] Practice Committee of the American Society for Reproductive Medicine (2012). Evaluation and treatment of recurrent pregnancy loss: A committee opinion. Fertil. Steril..

[7] Giorlandino C., Calugi G., Iaconianni L., Santoro M.L., Lippa A. (1998). Spermatozoa with chromosomal abnormalities may result in a higher rate of recurrent abortion. Fertil. Steril..

[8] Zidi-Jrah I., Hajlaoui A., Mougou-Zerelli S., Kammoun M., Meniaoui I., Sallem A., Brahem S., Fekih M., Bibi M., Saad A. (2016). Relationship between sperm aneuploidy, sperm DNA integrity, chromatin packaging, traditional semen parameters, and recurrent pregnancy loss. Fertil. Steril..

[9] Kiani-Esfahani A., Bahrami S., Tavalaee M., Deemeh M.R., Mahjour A.A., Nasr-Esfahani M.H. (2013). Cytosolic and mitochondrial ROS: Which one is associated with poor chromatin remodeling?. Syst. Biol. Reprod. Med..

[10] Kumar K., Deka D., Singh A., Mitra D.K., Vanitha B.R., Dada R. (2012). Predictive value of DNA integrity analysis in idiopathic recurrent pregnancy loss following spontaneous conception. J. Assist. Reprod. Genet..

[11] Venkatesh S., Kumar R., Deka D., Deecaraman M., Dada R. (2011). Analysis of sperm nuclear protein gene polymorphisms and DNA integrity in infertile men. Syst. Biol. Reprod. Med..

[12] Bronet F., Martinez E., Gaytan M., Linan A., Cernuda D., Ariza M., Nogales M., Pacheco A., San Celestino M., Garcia-Velasco J.A. (2012). Sperm DNA fragmentation index does not correlate with the sperm or embryo aneuploidy rate in recurrent miscarriage or implantation failure patients. Hum. Reprod..

[13] Bellver J., Meseguer M., Muriel L., Garcia-Herrero S., Barreto M.A., Garda A.L., Remohi J., Pellicer A., Garrido N. (2010). Y chromosome microdeletions, sperm DNA fragmentation and sperm oxidative stress as causes of recurrent spontaneous abortion of unknown etiology. Hum. Reprod..

[14] Mohanty G., Jena S.R., Nayak J., Kar S., Samanta L. (2020). Proteomic Signatures in Spermatozoa Reveal the Role of Paternal Factors in Recurrent Pregnancy Loss. World J. Men’s Health.

[15] Naglot S., Tomar A.K., Singh N., Yadav S. (2021). Label-free proteomics of spermatozoa identifies candidate protein markers of idiopathic recurrent pregnancy loss. Reprod. Biol..

[16] Thapliyal A., Tomar A.K., Chandra K.B., Naglot S., Dhiman S., Singh N., Sharma J.B., Yadav S. (2023). Differential Sperm Proteomics Reveals the Significance of Fatty Acid Synthase and Clusterin in Idiopathic Recurrent Pregnancy Loss. Reprod. Sci..

[17] Ostermeier G.C., Miller D., Huntriss J.D., Diamond M.P., Krawetz S.A. (2004). Reproductive biology: Delivering spermatozoan RNA to the oocyte. Nature.

[18] Champroux A., Cocquet J., Henry-Berger J., Drevet J.R., Kocer A. (2018). A Decade of Exploring the Mammalian Sperm Epigenome: Paternal Epigenetic and Transgenerational Inheritance. Front. Cell Dev. Biol..

[19] Patronia M.-M., Potiris A., Mavrogianni D., Drakaki E., Karampitsakos T., Machairoudias P., Topis S., Zikopoulos A., Vrachnis D., Moustakli E. (2024). The Expression of microRNAs and Their Involvement in Recurrent Pregnancy Loss. J. Clin. Med..

[20] Alipour M., Abtin M., Hosseinzadeh A., Maleki M. (2019). Association between miR-146a C>G, miR-149 T>C, miR-196a2 T>C, and miR-499 A>G polymorphisms and susceptibility to idiopathic recurrent pregnancy loss. J. Assist. Reprod. Genet..

[21] Zhang Y., Zhou J., Li M.Q., Xu J., Zhang J.P., Jin L.P. (2019). MicroRNA-184 promotes apoptosis of trophoblast cells via targeting WIG1 and induces early spontaneous abortion. Cell Death Dis..

[22] Dong X., Yang L., Wang H. (2017). miR-520 promotes DNA-damage-induced trophoblast cell apoptosis by targeting PARP1 in recurrent spontaneous abortion (RSA). Gynecol. Endocrinol..

[23] Yang Q., Gu W.W., Gu Y., Yan N.N., Mao Y.Y., Zhen X.X., Wang J.M., Yang J., Shi H.J., Zhang X. (2018). Association of the peripheral blood levels of circulating microRNAs with both recurrent miscarriage and the outcomes of embryo transfer in an in vitro fertilization process. J. Transl. Med..

[24] Wang J.M., Gu Y., Zhang Y., Yang Q., Zhang X., Yin L., Wang J. (2016). Deep-sequencing identification of differentially expressed miRNAs in decidua and villus of recurrent miscarriage patients. Arch. Gynecol. Obstet..

[25] Bruno V., Amati F., Ticconi C., Riccio S., Vancheri C., Rizzacasa B., Splendiani E., Ferretti E., Ernerudh J., Piccione E. (2022). Low molecular weight heparin -induced miRNA changes in peripheral blood mononuclear cells in pregnancies with unexplained recurrent pregnancy loss. J. Reprod. Immunol..

[26] Yamauchi Y., Shaman J.A., Ward W.S. (2011). Non-genetic contributions of the sperm nucleus to embryonic development. Asian J. Androl..

[27] Schagdarsurengin U., Paradowska A., Steger K. (2012). Analysing the sperm epigenome: Roles in early embryogenesis and assisted reproduction. Nat. Rev. Urol..

[28] Davies R., Jayasena C.N., Rai R., Minhas S. (2023). The Role of Seminal Oxidative Stress in Recurrent Pregnancy Loss. Antioxidants.

[29] Jena S.R., Nayak J., Kumar S., Kar S., Dixit A., Samanta L. (2021). Paternal contributors in recurrent pregnancy loss: Cues from comparative proteome profiling of seminal extracellular vesicles. Mol. Reprod. Dev..

[30] Imam S.N., Shamsi M.B., Kumar K., Deka D., Dada R. (2011). Idiopathic recurrent pregnancy loss: Role of paternal factors; a pilot study. J. Reprod. Infertil..

[31] du Fosse N., van der Hoorn M.L., Eikmans M., Heidt S., le Cessie S., Mulders A., van Lith J., Lashley E. (2019). Evaluating the role of paternal factors in aetiology and prognosis of recurrent pregnancy loss: Study protocol for a hospital-based multicentre case-control study and cohort study (REMI III project). BMJ Open.

[32] Xue D., Zhang Y., Wang Y., Wang J., An F., Sun X., Yu Z. (2019). Quantitative proteomic analysis of sperm in unexplained recurrent pregnancy loss. Reprod. Biol. Endocrinol. RBE.

[33] Miller D., Ostermeier G.C. (2006). Towards a better understanding of RNA carriage by ejaculate spermatozoa. Hum. Reprod. Update.

[34] Wang Y., Liu H.Z., Liu Y., Wang H.J., Pang W.W., Zhang J.J. (2018). Downregulated MALAT1 relates to recurrent pregnancy loss via sponging miRNAs. Kaohsiung J. Med. Sci..

[35] Abu-Halima M., Hammadeh M., Schmitt J., Leidinger P., Keller A., Meese E., Backes C. (2013). Altered microRNA expression profiles of human spermatozoa in patients with different spermatogenic impairments. Fertil. Steril..

[36] Abu-Halima M., Hammadeh M., Backes C., Fischer U., Leidinger P., Lubbad A.M., Keller A., Meese E. (2014). Panel of five microRNAs as potential biomarkers for the diagnosis and assessment of male infertility. Fertil. Steril..

[37] Wang C., Yang C., Chen X., Yao B., Yang C., Zhu C., Li L., Wang J., Li X., Shao Y. (2011). Altered profile of seminal plasma microRNAs in the molecular diagnosis of male infertility. Clin. Chem..

[38] Wu C., Blondin P., Vigneault C., Labrecque R., Sirard M.A. (2020). Sperm miRNAs-potential mediators of bull age and early embryo development. BMC Genom..

[39] Cao W., He Y., Lan J., Luo S., Sun B., Xiao C., Yu W., Zeng Z., Lei S. (2024). FOXP3 promote the progression of glioblastoma via inhibiting ferroptosis mediated by linc00857/miR-1290/GPX4 axis. Cell Death Dis..

[40] Hekim N., Gunes S., Ergun S., Barhan E.N., Asci R. (2024). Investigation of sperm hsa-mir-145-5p and MLH1 expressions, seminal oxidative stress and sperm DNA fragmentation in varicocele. Mol. Biol. Rep..

[41] Zhao M.J., Zhang Y.N., Zhao Y.P., Chen X.B., Han B.S., Ding N., Gu Y.Q., Wang S.S., Ma J., Liu M.L. (2023). Altered microRNA expression profiles of human spermatozoa in normal fertile men of different ages. Asian J. Androl..

[42] Wu J., Bao J., Kim M., Yuan S., Tang C., Zheng H., Mastick G.S., Xu C., Yan W. (2014). Two miRNA clusters, miR-34b/c and miR-449, are essential for normal brain development, motile ciliogenesis, and spermatogenesis. Proc. Natl. Acad. Sci. USA.

[43] Wu Z., Wang H., Fang S., Xu C. (2015). MiR-449c inhibits gastric carcinoma growth. Life Sci..

[44] Sandbothe M., Buurman R., Reich N., Greiwe L., Vajen B., Gurlevik E., Schaffer V., Eilers M., Kuhnel F., Vaquero A. (2017). The microRNA-449 family inhibits TGF-beta-mediated liver cancer cell migration by targeting SOX4. J. Hepatol..

[45] Wang X., Guo S., Zhou X., Wang Y., Zhang T., Chen R. (2021). Exploring the Molecular Mechanism of lncRNA-miRNA-mRNA Networks in Non-Syndromic Cleft Lip with or without Cleft Palate. Int. J. Gen. Med..

[46] Yuan P., Wang Z.H., Jiang H., Wang Y.H., Yang J.Y., Li L.M., Wang W.T., Chen J., Li D.H., Long S.Y. (2024). Prevalence and plasma exosome-derive microRNA diagnostic biomarker screening of adolescent idiopathic scoliosis in Yunnan Province, China. Front. Pediatr..

[47] Kovacs C.S. (2015). Calcium, phosphorus, and bone metabolism in the fetus and newborn. Early Hum. Dev..

[48] Whitaker M. (2008). Calcium signalling in early embryos. Philos. Trans. R. Soc. Lond. Ser. B Biol. Sci..

[49] Wang W., Fu S., Lin X., Zheng J., Pu J., Gu Y., Deng W., Liu Y., He Z., Liang W. (2019). miR-92b-3p Functions As A Key Gene In Esophageal Squamous Cell Cancer As Determined By Co-Expression Analysis. OncoTargets Ther..

[50] Du Y., Miao Z., Wang K., Lv Y., Qiu L., Guo L. (2021). Expression levels and clinical values of miR-92b-3p in breast cancer. World J. Surg. Oncol..

[51] Long M., Zhan M., Xu S., Yang R., Chen W., Zhang S., Shi Y., He Q., Mohan M., Liu Q. (2017). miR-92b-3p acts as a tumor suppressor by targeting Gabra3 in pancreatic cancer. Mol. Cancer.

[52] Zhao F., Yang Z., Gu X., Feng L., Xu M., Zhang X. (2021). miR-92b-3p Regulates Cell Cycle and Apoptosis by Targeting CDKN1C, Thereby Affecting the Sensitivity of Colorectal Cancer Cells to Chemotherapeutic Drugs. Cancers.

[53] Ma H., Wang L.Y., Yang R.H., Zhou Y., Zhou P., Kong L. (2019). Identification of reciprocal microRNA-mRNA pairs associated with metastatic potential disparities in human prostate cancer cells and signaling pathway analysis. J. Cell. Biochem..

[54] Zeng C., Duan S., Zhao L., Jiang J. (2024). Hsa-miR-92b-3p Targeting FHL2 to Enhance Radiosensitivity of Nasopharyngeal Carcinoma. Biochem. Genet..

[55] Heidary Z., Zaki-Dizaji M., Saliminejad K., Khorram Khorshid H.R. (2019). MicroRNA profiling in spermatozoa of men with unexplained asthenozoospermia. Andrologia.

[56] von Grothusen C., Frisendahl C., Modhukur V., Lalitkumar P.G., Peters M., Faridani O.R., Salumets A., Boggavarapu N.R., Gemzell-Danielsson K. (2022). Uterine fluid microRNAs are dysregulated in women with recurrent implantation failure. Hum. Reprod..

[57] Polytarchou C., Hatziapostolou M., Yau T.O., Christodoulou N., Hinds P.W., Kottakis F., Sanidas I., Tsichlis P.N. (2020). Akt3 induces oxidative stress and DNA damage by activating the NADPH oxidase via phosphorylation of p47(phox). Proc. Natl. Acad. Sci. USA.

[58] Nonoguchi K., Tokuchi H., Okuno H., Watanabe H., Egawa H., Saito K., Ogawa O., Fujita J. (2001). Expression of Apg-1, a member of the Hsp110 family, in the human testis and sperm. Int. J. Urol..

[59] Son W.Y., Han C.T., Hwang S.H., Lee J.H., Kim S., Kim Y.C. (2000). Repression of hspA2 messenger RNA in human testes with abnormal spermatogenesis. Fertil. Steril..

[60] Erata G.O., Kocak Toker N., Durlanik O., Kadioglu A., Aktan G., Aykac Toker G. (2008). The role of heat shock protein 70 (Hsp 70) in male infertility: Is it a line of defense against sperm DNA fragmentation?. Fertil. Steril..

[61] Hu Y., Zhou Z., Huang X., Xu M., Lu L., Xu Z., Li J., Sha J. (2004). Expression of a novel DnaJA1 alternative splicing in human testis and sperm. Int. J. Androl..

[62] Tang W., Liu D.F., Kai H., Zhao L.M., Mao J.M., Zhuang X.J., Ma L.L., Hui J. (2015). miRNA-mediated regulation of heat shock proteins in human ejaculated spermatozoa. Turk. J. Med. Sci..

[63] Chang S., Yin T., He F., Ding J., Shang Y., Yang J. (2020). CaMK4 promotes abortion-related Th17 cell imbalance by activating AKT/mTOR signaling pathway. Am. J. Reprod. Immunol..

[64] Moreno R.D., Urriola-Munoz P., Lagos-Cabre R. (2011). The emerging role of matrix metalloproteases of the ADAM family in male germ cell apoptosis. Spermatogenesis.

[65] Shafiq T.A., Yu J., Feng W., Zhang Y., Zhou H., Paulo J.A., Gygi S.P., Moazed D. (2024). Genomic context- and H2AK119 ubiquitination-dependent inheritance of human Polycomb silencing. Sci. Adv..

[66] Li M.W., Mruk D.D., Cheng C.Y. (2009). Mitogen-activated protein kinases in male reproductive function. Trends Mol. Med..

[67] Huang C., Liu W., Ji G.X., Gu A.H., Qu J.H., Song L., Wang X.R. (2012). Genetic variants in TP53 and MDM2 associated with male infertility in Chinese population. Asian J. Androl..

[68] Nakayama Y., Yamamoto T., Abe S.I. (1999). IGF-I, IGF-II and insulin promote differentiation of spermatogonia to primary spermatocytes in organ culture of newt testes. Int. J. Dev. Biol..

[69] Raimondo S., Gentile T., Gentile M., Morelli A., Donnarumma F., Cuomo F., De Filippo S., Montano L. (2019). p53 Protein Evaluation on Spermatozoa DNA in Fertile and Infertile Males. J. Hum. Reprod. Sci..

[70] Selvaraju S., Parthipan S., Somashekar L., Kolte A.P., Krishnan Binsila B., Arangasamy A., Ravindra J.P. (2017). Occurrence and functional significance of the transcriptome in bovine (*Bos taurus*) spermatozoa. Sci. Rep..

[71] Kierszenbaum A.L. (2006). Tyrosine protein kinases and spermatogenesis: Truncation matters. Mol. Reprod. Dev..

[72] Xu W.M., Chen J., Chen H., Diao R.Y., Fok K.L., Dong J.D., Sun T.T., Chen W.Y., Yu M.K., Zhang X.H. (2011). Defective CFTR-dependent CREB activation results in impaired spermatogenesis and azoospermia. PLoS ONE.

[73] Bains R., Adeghe J., Carson R.J. (2002). Human sperm cells express CD44. Fertil. Steril..

[74] Ickowicz D., Finkelstein M., Breitbart H. (2012). Mechanism of sperm capacitation and the acrosome reaction: Role of protein kinases. Asian J. Androl..

[75] Angeles-Floriano T., Roa-Espitia A.L., Baltierrez-Hoyos R., Cordero-Martinez J., Elizondo G., Hernandez-Gonzalez E.O. (2016). Absence of aryl hydrocarbon receptor alters CDC42 expression and prevents actin polymerization during capacitation. Mol. Reprod. Dev..

[76] Asadi A., Ghahremani R., Abdolmaleki A., Rajaei F. (2021). Role of sperm apoptosis and oxidative stress in male infertility: A narrative review. Int. J. Reprod. Biomed..

[77] Chhikara N., Tomar A.K., Datta S.K., Yadav S. (2023). Proteomic changes in human spermatozoa during in vitro capacitation and acrosome reaction in normozoospermia and asthenozoospermia. Andrology.

[78] Friedlander M.R., Mackowiak S.D., Li N., Chen W., Rajewsky N. (2012). miRDeep2 accurately identifies known and hundreds of novel microRNA genes in seven animal clades. Nucleic Acids Res..

[79] Robinson M.D., Oshlack A. (2010). A scaling normalization method for differential expression analysis of RNA-seq data. Genome Biol..

[80] Sticht C., De La Torre C., Parveen A., Gretz N. (2018). miRWalk: An online resource for prediction of microRNA binding sites. PLoS ONE.

[81] Shannon P., Markiel A., Ozier O., Baliga N.S., Wang J.T., Ramage D., Amin N., Schwikowski B., Ideker T. (2003). Cytoscape: A software environment for integrated models of biomolecular interaction networks. Genome Res..

[82] Szklarczyk D., Kirsch R., Koutrouli M., Nastou K., Mehryary F., Hachilif R., Gable A.L., Fang T., Doncheva N.T., Pyysalo S. (2023). The STRING database in 2023: Protein-protein association networks and functional enrichment analyses for any sequenced genome of interest. Nucleic Acids Res..

[83] Xie S., Zhu Q., Qu W., Xu Z., Liu X., Li X., Li S., Ma W., Miao Y., Zhang L. (2019). sRNAPrimerDB: Comprehensive primer design and search web service for small non-coding RNAs. Bioinformatics.

