# Supplementary material for: Exploring Differentially Expressed Sperm miRNAs in Idiopathic Recurrent Pregnancy Loss and Their Association with Early Embryonic Development
Source: Noncoding RNA. 2024 Jul 21;10(4):41. doi: 10.3390/ncrna10040041 (PMC11270218; doi:10.3390/ncrna10040041)
Supplement: Supplementary file 1 [file ncrna-10-00041-s001.zip › Table S1.pdf]

Table S1: List of primers

**Mature miRNA name**	**Sequence (5'-->3')**	**Tm (ºC)**
hsa-miR-92b-3p	AACAATTATTGCACTCGTCCCGG	53
hsa-miR-7977	AACAAGTTCCCAGCCAACGC	56
hsa-miR-1246	AACACGCAATGGATTTTTGGAGC	55
hsa-miR-1290	AACACGTGTGGATTTTTGGATCAGG	58
hsa-miR-145-5p	AACACGCGTCCAGTTTTCCC	61
hsa-miR-142-3p	AAGAGCGTTGTAGTGTTTCCTACTT	55
hsa-miR-449c-5p	AACAGATAGGCAGTGTATTGCTAGC	55
hsa-miR-4454	AACAAGGGATCCGAGTCACGG	59
hsa-miR-29c-3p	AACAAGTAGCACCATTTGAAATCGG	59
hsa-miR-30b-5p	AACACGCTGTAAACATCCTACACTC	56
hsa-miR-519a-2-5p	AACACGTGCCTCTACAGGGAAG	56
hsa-miR-520b-5p	AACACGTGCCTCTACAGGGAAG	56
Reference gene (U6)	Forward: GGAACGATACAGAGAAGATTAGCA<br>Reverse: GTGCAGGGTCCGAGGT	53
